# Bibliographical Notices

**Published:** 1841-04-01

**Authors:** 


### Periscope;

OR,

### CIRCUMSPECTIVE REVIEW.

" Ore trahit quodcunque potest, atque addit acervo."

Descriptive Catalogue of the Preparations in the Museum of the Royal College of Surgeons in Ireland. By *John Houston*, M.D. M.R.I.A. Curator of the Museum. Vol. II. Pathology, pp. 604. Dublin, Hodges and Smith: London, Renshaw, 1840.

Mr. Houston is so favourably known to the profession, on this side of the water as well as on his own, that we need not say any thing complimentary about him. Yet the work before us deserves a compliment, for it is drawn up with care, judgment, and ability. It possesses, too, a wider sphere of utility than might be imagined from its name, for not only is it a Descriptive Catalogue, but its histories of cases, and accounts of pathological facts are, many of them, highly instructive.

The preparations in the museum and the descriptions of them here are arranged, not inconveniently, in six classes.

The first class, distinguished by the letter A, contains all the organs concerned in the assimilation of food.

The second, by B, contains the organs of circulation.

The third, by C, the organs of respiration.

The fourth, by D, the organs of sense.

The fifth, by E, the organs of locomotion and prehension.

The sixth, by F, the organs of generation and secretion of urine.

We cannot analyse, it would be impertinent to review, and we will not pretend to cull all the valuable facts out of this volume. It is one for the closet, adapted for reference, valuable for study. The student, young or old, who will take it into the museum it belongs to, and meditate upon the preparations on *those* shelves, their records in *these* pages, will have a large addition to his scientific knowledge, and thank Mr. Houston for his aid. Nay, the fire-side student who will take the book upon his knee, and carefully con it without the accompaniments of shelf or bottle, will rise from its perusal stored with much information that he could not be previously possessed of.

We will quote some of the facts which crowd the pages of the Catalogue, and which may seem curious, entertaining or instructive. But for every fact we *do* quote, our readers must understand that a hundred just as good remain behind. It would turn this Journal to a catalogue to do justice to all.

### *Face of a Man eaten by a Pig.*

A. *a*. 5.—Picture of a man whose face was eaten away, by a pig, while lying in a state of drunkenness. The entire nose, both cheeks, and parts of both ears, in fact, all the most eatable parts of his face were chewed off by the animal; nevertheless, the wounds all healed, and he recovered: but of course with all the disabilities of enunciation, chewing, and swallowing, attendant on such extensive destruction of soft parts. He, notwithstanding, under generous regimen contrived, while in Hospital, to keep up a good condition of body. His principal regret lay in the unavoidable disuse of his tobacco-pipe. The picture exhibits him after the wounds had all healed, without outward nose or ears, but with two beautifully white and perfect rows of teeth.

*Cancer of the Upper Lip.*

It is a curious circumstance that M. Cruveilhier denies positively the existence of cancer in the *upper* lip. He says, in his Descriptive Anatomy,—" Une particularité, tout inexplicable, c'est que les boutons cancéreux des lévres qui sont si fréquens, ne se remarquent jamais à la lévre supericure, mais toujours à la lévre inférieure." However true it may be that cancer is very much more frequent in the lower lip than in the upper, it certainly is *not* true that it is exclusively limited to the former. The following statements are conclusive on this point.

A. *a.* 9.—Incipient cancer of the upper lip, removed from a woman, seventy-one years of age. The disease appeared, at the time of the operation, to be local, but the patient died about a year and a half after, from its revival in the glands of the neck, without any recurrence of it in the lip.

A. *a.* 10.—Cancer engaging the right side of the *upper* lip, leaving the angle free. The patient was a tall, thin, unmarried woman, about sixty years of age. There is no report of the result of the operation.

A. *a.* 12.—Ulcerated cancer of the angle of the mouth, engaging equally both upper and lower lip. A flattened fungus may be seen on the inner surface, at the angle.

*Extirpation of Healthy Tongues.*

A. *a.* 35.—Two healthy tongues extirpated by evil-disposed persons, during the lifetime of the sufferers, with a view of preventing their giving evidence at a criminal trial.

A gang of ruffians waylaid the unfortunate men, threw them down, kneeled on their chests, and squeezed their throats, so as to make their tongues protrude from their mouths. This being effected, the tongues were laid hold of, pulled forcibly forwards, and cut out from near the root, by a short, sharp, curved weapon, like a gardener's knife. The victims of this atrocious deed recovered without ligatures to stop bleeding, or other special surgical treatment; and regained, afterwards, sufficient power of speech to convict and bring to punishment their assailants.

*Perforation of the Œsophagus by a Bougie.*

A. *a.* 44.—Stricture of the œsophagus behind the pericardium. The tube is narrowed to the extent of several inches, its walls at this place are thickened, and its mucous membrane corrugated longitudinally: in some parts there is a depressed, ulcerated surface; in others fungous projections. About midway between the cricoid cartilage and strictured part, there is a distinct oval ulcer, with spongy surface and fungous edges.

The history of this preparation is instructive. For a long period, the occasional introduction of the bougie gave relief; but finally, the operation became injurious; as on the last occasion, the bougie perforated the central ulcer observable in the preparation, and passed into the posterior mediastinum. From that moment the patient sank, and died in a few hours.

There might be many tallies found to this preparation and this history. Patients have been not unfrequently dispatched to " the undiscovered country " by œsophagus bougies. Surgeons should be chary of their use.

*Perforation of the Subclavian Artery by a Bone in the Œsophagus.*

A. *a.* 47.—The œsophagus of an old beggar-woman, stuffed with fragments of beef and bone, which caused death by suffocation. The sharp point of the bone having pierced the tunics of the œsophagus, wounded the right subclavian artery, which taking origin from the left part of the arch of the aorta, and crossing thence to the right, between the œsophagus and spine, happened to lie in the way. The projection of this artery may have even been, in part, a cause of the detention of the foreign body in the tube.

*Salivary Calculus Extracted.*

A. *a*. 85.—Salivary calculus, about one inch long and the thickness of a quill. It is slightly curved, and uniformly large from end to end. It was extracted from the submaxillary duct of a female, twenty-four years old. One end appeared at the mouth of the duct, so as to admit of being seized by a forceps. It came out easily. The patient was only inconvenienced afterwards by stiffness, and pain in moving the tongue. It is several years since the removal of the calculus, and no further complaint arising out of it has been made.

*Singular Atrophy of the Stomach.*

A. *b*. 93.—A portion of stomach in a singular state of atrophy. The organ, though much enlarged, contained only air; it had rejected all sorts of food. It was so thin in some parts, as to be almost transparent. The part preserved may give some idea of the state of the entire organ. The muscular coat appears to be that most reduced; the villi of the mucous surface, though very indistinct, are discernible; the serous coat is the most perfect. The patient was a lady of about sixty-five, of full, bloated person, though extremely temperate in habits. For a time she laboured under all the rational symptoms of thoracic aneurism, or incipient hydrothorax, puzzling many skilful physicians. Some months previous to her death, she had continual, and unappeasable hiccough, nausea and vomiting: she could not take food, great difficulty of breathing set in, and she died exhausted. The heart was found attenuated, flabby, and soft; and there were several osseous deposits, in the larger arteries.

*Cancer of the Stomach.*

A variety of preparations of cancer of the stomach are described. On the whole, they prove the too frequent obscurity of the symptoms attending that melancholy disease. If we might be allowed to generalise from what we have seen, we should say that medullary fungus of the stomach is less frequently attended with decided symptoms than scirrhus of it. The following case, as *a* case, countenances that idea.

A. *b*. 120.—Huge fungus in the stomach, springing from the greater curvature. The other parts of the organ are sound: it remains of the natural size; and the pylorus is free from disease. This was the case of a lunatic, of middle age, who enjoyed an excellent appetite; was not particutarly emaciated; and never made any complaint, leading to a suspicion of the stomach being diseased.

*Dangers of the Bougie.*

We are tempted again to allude to these, for the purpose of enjoining caution in the use of the œsophagus bougie—an instrument which, we fear, has been sadly abused. We cite another instance of a false route taken by it.

A. *b*. 146.—Enormous fungus of the stomach protruding in part, into the œsophageal orifice. Food could not pass the obstruction; and a bougie attempted to be introduced, opened a way into the peritoneum behind the fungus. The natural line of direction of the œsophagus with the stomach is completely altered. The œsophagus above the point of obstruction, is dilated and thinned. A bougie points out the course which the instrument took.

*Stomach of an Ironmonger.*

With an account of this we shall stop. The case, indeed, has been published, but may be new to some of our readers.

A. *b*. 188.—Stomach of a lunatic, in which were discovered various metallic substances; such as, the rusty remains of large nails, long pieces of thin iron-like portions of iron hoop, the worn-down blade of a knife, a large iron buckle, with a pewter tongue, as that of a saddle-stirrup, an iron hinge of a box or door, and several pieces of metal, too thin and worn to admit of their original use or form being in any way recognised. Four or five pieces in the same state, were also found in different parts of the intestinal canal. One, in particular, a piece of iron, four or five inches in length, lay in the transverse arch of the colon. On opening the abdomen in the first instance, the hollow viscera all presented an unusual dark tinge; and their fluid contents were of the same colour. There was no peritonitis. The preparation shews the stomach to be of inordinate size, and greatly thickened, both in its mucous and muscular tunics. The mucous glands are likewise hypertrophied; in size they resemble those in the crop of some granivorous birds, more than those of the human stomach. The rugæ, especially in the pyloric third of the stomach, are unusually prominent, being elevated into thick, firm, vascular masses, like fungous growths. There is at one particular spot, an ulcer with hardened edges. The œsophageal and pyloric orifices are both very much dilated.

If we close our notice of this volume here, it is rather because it admits of almost indefinite extension, than because we have exhausted its stock of interest and instruction. The profession may judge from what we have given, the value of what is behind, and we can say sincerely that the work is one fraught with information to those who will peruse it with attention. We repeat what we have before observed, that its utility is eminent as a book of reference.

---

NUCES PHILOSOPHICÆ; or the Philosophy of Things, as developed from the Study of the Philosophy of Words. By *Edward Johnson*, Esq. No. I. January, 1841. Price One Shilling. Simpkin and Co.

This periodical is dedicated to the members of the Provincial Medical Association, and is evidently the production of a clever and well-read man, who has studied philology with great assiduity. The nature of the work, however, is not sufficiently developed in this number to enable us to judge accurately of the performance. The dedication is very ingenious, and, we would say, rather too forensic. Indeed we think that Mr. Johnson would have figured at the bar, where he would have been able to make the " worse appear the better cause." In the present instance he has laboured hard, and with very specious and plausible arguments to prove that the study and practice of the medical art and science require a greater breadth and depth of intellectual power than that of either law or divinity. This may be, and probably is true. But the prudence or propriety of strongly urging comparisons, which are proverbially odious, may be well questioned, as coming from the acknowledged pen of a medical writer. Mr. Johnson has been hard upon the lawyer in particular; and we fear he has not been quite justified in considering that the only faculty of the mind called into operation by the lawyer is—MEMORY.

" And what are the intellectual attainments necessary to qualify the law-student for the exercise of his profession? To have (if it were possible) the *statutes at large* by heart—to store his memory with acts of parliament, and all such other writings as define the laws—and to have on the tip of his tongue, ready on all occasions, to hang a thief or save a thief, the previous decisions of previous judges which can be brought to bear on doubtful cases. Here again the faculty of the mind called into operation is *still the memory only*. The knowledge of the most profound lawyer consists solely in his recollection of the *opinions and writings* of other men, whether in the shape of acts of parliament or of adjudged cases—or rather, it is not knowledge at all—it is but fallible *opinion,* and consequently may be, and much of it has already been, *proved to be false.* To the law-student also, three years are, as I believe, the allotted period of study. Nor am I questioning either the propriety of this term, or the necessity of law-learning. I am only showing that it does not require a high order of intellect to master it, nor any large endowment of philosophical knowledge to become a profound lawyer." xi.

Whoever has listened to the orations of a Curran, a Brougham, a Romilly, or any other eminent barrister, and heard him range through the fields of literature and science for arguments, illustrations, and decorations, would candidly confess that a mere technical memory of the "statutes at large," could go but a short way in supplying the orator with materials for the defence of his own client, or the crimination of that of his antagonist.

That the passage above quoted, should have brought a hornet's nest about Mr. Johnson's ears, might have been readily anticipated—and accordingly his "philosophical nuts" have been cracked with sledge hammers by the legal tribe. We do not think that the policy of the provocation can be at all defended; but as the publication will be inevitably injured, if not strangled by the lawyers, while the divines will assist in the work of destruction, it behoves the medical profession to patronise the work, and carry it safely through the storm. For, although we cannot help considering the *comparison* which the author has drawn in favour of the medical profession, as injudicious; yet, it is clear that it was well-meant, and consequently entitled to our sympathy and support. We therefore solicit the patronage of the medical public in favour of this new periodical.

---

### GAS-BATHS.*

These and the Mud-baths to be presently described, are becoming very fashionable in Germany. From every inch of surface in the peat bog around Franzensbad, carbonic acid gas is constantly issuing forth in such quantities that its extrication is audible and visible, wherever there is water on the ground. To have a reservoir of this gas, it is only necessary to build a house, and prevent the carbonic acid from being dispersed in the air. It is there collected, and baths and douches are constructed for its ready application to the body generally, or to any particular part thereof. The gas-bath or building at Franzensbad, stands within thirty or forty yards of the Franzensquelle, and from the ground of this house, which is of very moderate extent, there issue 5760 cubic *feet* of gas every twenty-four hours!! There is little doubt that the extrication of carbonic acid is equally plentiful in any and every part of the bog in which the town is situated. I should think that to go to sleep on the ground, in a calm Summer's night, would be inevitable death. As it is, the good people of Franzensbad, inhabitants and visitors, must be perpetually inhaling an atmosphere well impregnated with this gas. I do not suppose, however, that this is productive of any injurious effects.

The gas is conveyed into the bath through a cock at the bottom, and the patient, being either dressed or undressed, sits down on a little stool, while a wooden lid or cover, with a hole that fits tolerably close to the neck, is placed over the body, the head being in the open air. They have small tubes through which they can apply the gas to the eyes, ears, or any part of the body, in a stream, the velocity of which can be augmented or diminished at pleasure.

---

* From Dr. Johnson's "Pilgrimages to the Spas in Pursuit of Health."

They can also diminish the intensity of the gas by applying a piece of muslin or taffeta over the pipe, or over the eyes or ears that are subjected to the stream. I did not try the gas baths here, but at Marienbad I used them generally and locally, accompanied by my kind friend Dr. Herzig of that place. Standing in the bath, the cock was turned without my being aware of it, and, in a few seconds, I felt a sense of heat ascending quickly along my legs towards the body. Without thinking of the gas I stooped, and put my head down to the aperture of the tube, by which I inhaled as much of the carbonic acid as caused a sudden faintness. Dr. H. and the bathman quickly extricated me from my perilous situation, and I went on with the bath, while my head was in the open air. I found that the following representation of the sensible, and physiological effects of the bath, as given by Baron Aimé, is sufficiently correct. 1. The gas excites and even irritates the skin, producing a pricking, and soon afterwards a strong itching on the surface, accompanied by heat, and ultimately perspiration. 2. The gas stimulates the nerves of all parts to which it is applied. [I had a stream directed on my eyes, which caused a most profuse flow of tears, with strong sense of heat. When it was applied to my ears, a sense of heat, and a considerable noise were the effects produced.*] 3. It is asserted by physicians of the Continent that this gas is extremely useful when applied to old, ill-conditioned, and irritable ulcers, as soothing and promotive of healthy discharge, and ultimately of cicatrisation. 4. Although the breathing of this gas is as mortal as that of the Grotto de Cane, yet if diluted with plenty of atmospheric air, it is thought that it might prove serviceable in some states or stages of phthisis, asthma, &c. 5. The action of this gas on the eyes and ears I have already mentioned. Its remedial agency is much extolled in certain disorders or diseases of those organs, attended with atony or morbid irritability of their nerves and structures. 6. These baths are chiefly employed in cases of paralysis attended with stiffness, feebleness, or spasmodic movements. 7. In chronic, inveterate affections of a gouty or rheumatic nature—chronic sores—glandular swellings—and various cutaneous complaints, the gas baths are applied, and, as is affirmed, with success. 8. In uterine affections, irregularities, &c. attended with torpor, debility, and irritability.

Upon the whole I am disposed to think that the gas baths are active agents, and that they may be made useful ones, when carefully applied.

### MUD-BATHS.†

Among the novelties—transcendentalisms, or, as some would call them, extravaganzas, of Germany, the MUD BATHS deserve the "passing tribute" of a short notice. But alas! there is "nothing new under the sun"—or under the earth. To the mud of the Nile and the Ganges, virtues almost miraculous—even the creative power of life—have been attributed, time immemorial. Who does not know that the life of MARIUS was preserved by a mud-bath in the Minturnian marshes?—The instincts of animals, too, are not to be overlooked. We all know the extreme tenacity of life possessed by eels—owing perhaps to their frequent use of mud-baths. Swine are proverbially subject to cutaneous complaints, especially measles; to prevent or cure which, Nature seems to prompt the daily employment of mud-baths, in the Summer season. A remarkable instance of the force of instinct is afforded by the Indian buffalo. That animal

* The Baron Aimé suggests the more frequent application of this gas to certain complaints of both sexes which are regarded with no small anxiety by both parties. *Verbum sat.*

† From Dr. Johnson's "Pilgrimages to the Spas in Pursuit of Health."

immerses himself daily, during the hot season, in mud, up to the very nose; by which means, we may conclude that he avoids the jungle fever, or cures himself of liver-complaints. The alligator offers another example. When he has swallowed a buffalo or a tiger, he buries himself up to the nose in mud, on the oozy shores of the Ganges, no doubt for the promotion of digestion.

It is unnecessary to multiply the virtues of mud-baths. Those who desire ocular proofs must repair to Franzensbad in Bohemia, where they will see—not mud but bog-baths in perfection; though they are now also got up very well in Marienbad, Carlsbad, Teplitz, and other fashionable spas.

I have alluded to the plentiful supply of bog which the immediate vicinity of Franzensbad offers to the mud-bathers. This earth contains the following materials:—viz. The fibres of plants not decomposed, and whose organization is recognizable—matters soluble in water, such as vegetable substances rich in carbon, and of a yellow colour;—sulphate of lime—sulphate of magnesia—sulphate of iron—alum—bituminous extractive matter—oxide of iron—fine sand.

Thus we see that the mere boggy material of the mud-bath contains many substances that may and do exercise a considerable physiological action on the body; and medicinal agency on the constitution.

The peat bog is carried to the neighbourhood of the baths, and there allowed to dry to some extent. It is then sifted and separated from the woody fibres and coarser materials, when it is mixed with the mineral water of the Salzenquelle into the consistence of a very soft poultice. In this state it is heated by steam to a temperature varying from 80° to 100° of Fahrenheit, when it is ready for the bather, being worked up by means of wooden instruments and the hands into a complete black amalgam. I took the mud-bath here, at Marienbad, and Carlsbad, and do not regret the experiments. I confess that, at first, I felt some repugnance, not fear, in plunging into the black peat poultice; but when up to the chin (temperature 97°) I felt more comfortable than I had ever done, even in the baths of Schlangenbad, Wildbad, or Pfeffers. The material is so dense, that you are some time in sinking to the bottom of the bath—and I could not help fancying myself in Mahomet's tomb, suspended between Heaven and Earth, but possessing consciousness, which I fear the prophet did not enjoy. There was one drawback on the mud-bath, or peat-poultice. We cannot roll about, like a porpoise or whale, as in the water-bath, without considerable effort, so dense is the medium in which we lie; but I found that I could use friction to all parts of the body, with great ease, in consequence of the unctuous and lubricating quality of the bath. After twenty minutes' immersion, I felt an excitement of the surface, quite different from that of the common mineral warm baths—even of those of Wisbaden, Kissengen—or Schwalbach—attended, as I fancied, by elevation of spirits.

Whilst I was thus philosophizing, like Diogenes, in my tub, the thought came across my mind that I would have a dive in the sable mixture. I knew that the sun and winds had so tanned my complexion, that IT would not suffer by immersion; and if my hair should get dyed black, the change would certainly be for the better. I therefore disappeared like an eel in the mud; but, on emerging from the bog, I thought I should have been suffocated before I cleared my face from the tenacious cataplasm. I had now been nearly half an hour in the Schlammbad, and prepared to quit, as the mixture was fast cooling down, and the heat could not be kept up, as in the water-bath. On raising myself slowly and perpendicularly, with at least twenty pounds of mud on my surface, I caught a full length portrait of myself in the glass, and I think the view would have sickened Narcissus of self-contemplation for ever!! I was really shocked at my sudden metamorphosis into the Œthiopian, and began to doubt whether I should ever "change my hue" again. The warm water-bath was close at hand, but I had the presence of mind not to jump into it at once, as I should, in that case, render it a black wash-tub; but by clearing away with both hands, some sixteen or eighteen pounds of peat varnish from my body, I rolled into the clear fluid, where it required half an hour's rubbing and scrubbing to purify myself from the "Bain de Boue." Both on this, and on subsequent occasions, at Marienbad, Carlsbad, and Teplitz, I experienced a degree of exhilaration, strength, and elasticity from the mud-bath, which I had never done from any other. The iron in these baths, instead of corrugating the skin, as I expected, imparts to it a glossy or sattiny feel and softness quite peculiar—and much more in degree than the waters of Schlangenbad.

The bog-earth is well picked, and in some places sifted, so as to remove all the fibrous and woody parts, leaving the fat unctuous substance to be mixed with the mineral water of the place. In general these baths produce a pricking sensation, and sometimes an eruption on the skin, an effect which I did not experience.* They are therefore much used in old and obstinate cutaneous complaints, as well as in glandular swellings, sequences of gout, rheumatism, &c. They are very exciting to the nervous system, and should not be used where there are any local inflammations, or much general excitability of the constitution. They do not lose their heat so rapidly as the water-baths, and consequently they maintain the volatile and penetrating principles longer than the latter. They are much employed in paralysis, chronic ulcers, and cutaneous affections.

Here and at other spas where mud-baths are employed, I met with several veteran warriors, whose aching wounds reminded them too often of battle-fields and bloody campaigns. They almost all agreed in attributing more efficacy to these than to the common baths—and I think, from what I have seen, heard, and felt, that there is much truth in these statements. The Schlammbads have one advantage over the others, which is more prized on the Continent than in England—the facilities which they afford the bathers, both male and female, of receiving morning visits from their friends while in the mud, and that without any violation of delicacy, propriety, or decorum; for there, persons are more completely veiled than in any dress, even of the most dense and sable furs of Russia. An English lady of rank, at Teplitz, was visited by her physician and friends while immersed to the chin in peat-bog. They read to her, and conversed with her till the signal was given for exchanging the black varnish for the limpid and purifying wave, when they retired.

The rules for taking the Franzensbad waters and baths do not vary materially from those of other spas. The following concise direction is from the pen of Dr. Clarus.

"A complete course of these waters requires at least four weeks. When it is thought desirable to take of more than one source, the change from one well to another should not be abrupt, but gradual. We may commence with one glass of the Salzquelle, and each day increase by the glass, till, in a week, we come to six or seven glasses, taken at intervals of a quarter of an hour. After this period, the Salzquelle is to be decreased, glass by glass, and replaced by the Cold Sprudel. This change is to go on during the second week. At the end of a fortnight, the Cold Sprudel is to be changed, in the same gradual manner, for the Franzensquelle, which is to be continued till the end of the course, unless some circumstances arise to alter the arrangement. Those who are of very weakly constitutions, and especially if they labour under any pulmonary complaint, will do well to add some warm milk or whey to the mineral water."

The baths are generally taken about two hours after breakfast. They ought not to be taken unless the bowels are daily opened, either by the waters or by aperient medicine. The temperature of the baths should be about 98° of Fahrenheit, or that of the blood.

---

* Dr. Clarus, Dr. Granville, and others state that the skin exhales an acid odour, and even feels salt to the tongue for several hours after leaving the bath. This I did not perceive in my own case at all.

Baron Aimé has collected from various sources a host of cases, of all kinds of maladies, cured or relieved by the waters of Franzensbad; but into these it is unnecessary to go. Here the tyrant fashion has caused a comparative desertion for the more attractive localities, if not more sanative springs, of Marienbad, Carlsbad, and Teplitz. The qualities of the mud, and the profusion of the gas, at Franzensbad, however, may probably turn the current by and bye in its favour.

---

PRACTICAL OBSERVATIONS ON DISTORTIONS OF THE SPINE, CHEST, AND LIMBS; TOGETHER WITH REMARKS ON PARALYTIC AND OTHER DISEASES CONNECTED WITH IMPAIRED OR DEFECTIVE MOTION. By *William Tilleard Ward*, F.L.S. Member of the Royal College of Surgeons, Fellow of the Royal Medical and Chirurgical Society, and Corresponding Fellow of the Medical Society of London. Renshaw, 1840.

This is a Second Edition. We shall not, therefore, notice it at length, but content ourselves with selecting a few points for observation.

Our author's mode of treatment may be said to hang on his physiological views, not perhaps very novel or striking, with respect to muscular power and perfection. That power he concludes to hinge:—

1. On the state of the functions of respiration and circulation, and that increased strength is a consequence of increased vascularity and circulation of blood in the part, and *vice versâ*, a want of tone and power, of a deficient supply of it.

2. On the degree of exercise, or frequency with which they are called into action.

3. On the mental energy, or power of volition exerted on them.

4. That the most effectual means of increasing muscular strength, is by the frequent exercise of the power itself, and, consequently, the preservation of the healthy actions of those functions by which it is influenced.

5. That the muscular parts have a constant tendency to contract, by which they adapt themselves to the state of the limb, or parts to which they are attached.

To muscular debility Mr. Ward attributes much in the production of spinal curvature, unattended with caries. The proportion of the various kinds of spinal curvature to one another is thus stated by our author from his own experience. Of two hundred and eighty-two cases of spinal curvature there were:—

| | |
|---|---|
| Of curvature to the right side without disease .. .. .. | 230 |
| Of curvature to the left side without disease.. .. .. .. | 10 |
| Of posterior curvature unaccompanied by disease.. .. .. | 9 |
| Of posterior curvature with disease .. .. .. .. .. .. | 30 |
| Of that which I denominate incurvation, *i. e.* projection of the lumbar vertebræ within the pelvis .. .. .. .. .. | 3 |

Mr. Ward attaches some degree of importance to the condition of the intervertebral substances. After noticing the fact of the diurnal loss of stature from compression of them, he goes on to observe, that the greater strength of the intervertebral substance in persons advanced in life, in connexion also with the muscular structure, may be assigned as the cause of their general exemption from this disorder; Mr. Pott having remarked that he had "never seen it at an age beyond forty." In youth the cartilages are less firm than in the adult age, and from a cursory review of the above experiments it would appear, that to the want of firmness of the intervertebral substance may be ascribed the occurrence of incurvation; if we, however, take into consideration the uses of the muscles, it will be seen, that when in the erect position, if they act in a natural manner, the cartilages will not be pressed upon in an undue direction, either laterally or anteriorly, so as to produce distortion, but that the only effect of the superincumbent weight will be for the time to decrease the height of the individual, by bringing the bodies of the vertebræ more closely together. On the recurrence to the horizontal position, the spine will resume its proper length. Less consequence has been attached to the influence of the cartilages and ligaments in the production of this disorder, than the importance of their functions to the motions of the spine may seem to demand. This has arisen, however, from observation both in health and disease, that their relaxation or firmness, increase or decrease of size, power or weakness, will be commensurate with the tone and vigour of the muscular structure; that the diminished strength of the ligaments and cartilages, is a sequel of muscular debility: and that, therefore, by giving power to the muscles, an accession of strength to the ligaments and intervertebral substance will result as a matter of course.

Our author thus describes what he calls *Incurvation of the Spine.*

"There is an affection of the spine consequent upon paralysis, that has not been noticed, that I am aware of, by any author: it consists in a falling down of the dorsal and lumbar vertebræ within the pelvis; it is not of very frequent occurrence; I have seen only three cases. It may be denominated incurvation of the spine. The lumbar vertebræ project anteriorly towards the pubis, causing a great hollowness of the back, and giving rise to a peculiar waddling gait in the person affected with the disorder.

In this particular form of distortion, in addition to the ordinary means, I have recommended that the patient should make use of chairs as little as possible, and rather sit on a couch, or on the ground, with the legs folded in the manner of the Turks, or as tailors are seated when at their usual employment."

Mr. Ward's plan of treatment consists in regulated diet—the exclusion of improper muscular habits—the horizontal posture—and judicious muscular exercises. He observes of the inclined plane—

"The inclined plane has been much used for the purpose of confining the body to a particular position, in the recumbent state, for a considerable length of time, without allowing any alteration of posture. This is extremely disagreeable to the patient, and sometimes productive of distressing feelings, without being compensated by any particular advantage that may not be gained by allowing rest on a mattress or sofa, without restraint on the motion of the body.

It has been remarked to me by patients themselves, as well as by those around them, that they arose from the former not only without feeling refreshed, but sometimes greatly fatigued; and if we reflect, that the alternations of position in the body throughout the day, and even during sleep, are so many changes to relieve the contractions of the muscles, and that in this confined attitude a great number of them must be kept in action during the time it is persevered in, and that they cannot support long continued exertion without great weariness supervening, we shall be at no loss to discover why such an effect should be produced."

A favourite exercise is this,—a weight appended to a cord is passed over a pulley, and the other extremity, having a strap attached to it, is fastened round the patient's head; the pelvis being fixed, the patient is directed to raise the weight by drawing the head and trunk backwards, and to repeat this effort until fatigue is produced. The frequency of repetition of this exercise of the muscles, and the weight of the body to be raised, must, of course, depend on the patient's strength. After each effort, it is advisable to take rest, by lying down on a couch, or sofa, in order that the muscles may not be placed on the stretch, and thus prevented from recovering themselves. This mode of exercising the muscles is equally applicable to the anterior curvature of the spine, when not connected with caries, as to that which takes place laterally.

Friction and Shampooing are also beneficial. He objects to the opinion entertained at times and given that the patient will "grow out" of her distortion. He has not found such predictions true. He adds with candour,—

"I beg here not to be understood, as wishing to assert, that I have in every instance effected a complete restoration of the spine to its proper shape in cases of lateral spinal curvature, unaccompanied by disease of the bone; a regard for truth compels me to admit, that in several cases of long standing, treated by me upon the plan before mentioned, and where, in addition to the above means, extension has been resorted to, I have failed; the curvature originally, perhaps, between an inch and a half and two inches, has been reduced to within a quarter of an inch, beyond which, an advance could not be made; although I have every reason to believe that my patients have strictly followed my injunctions in every respect, both with regard to exercise, and the strict observance of the recumbent posture. In these cases there was no suspicion of diseased bone; if there had been, I could not have used the same means (extension) without producing mischief. The average rate at which improvement takes place, presuming the curvature to be of the extent of an inch and a half or two inches, is, half an inch the first month, and one-eighth of an inch every succeeding two months. I have witnessed some cases, where the diminution of the curvature has proceeded more quickly; these are, however, rare, occurring either in natives of India, or very young subjects, or where the progress of the distortion has been unusually rapid."

*Deformity of the Chest.*—There is a chapter on this. The malformation termed "chicken-breast" is that more particularly dwelt on. We agree with Mr. Ward on the tendency of the chicken-breasted to pulmonary as well as to cardiac disease—on the gravity, therefore, of the affection. Passing over his theory of its causation, we come to his mode of treatment.

"The method which I have employed with regard to the local means in those cases, where the spine has been exempt from disease, has been that of placing the intercostal muscles and those connected with the anterior part of the chest on the stretch, by placing the patient in a standing position, with the back against a cylindrical piece of wood, and the arms extended backwards. By this means an extension of the pectoral muscles is produced, and they are thus brought into full action upon the ribs, as well as the muscles of the abdomen which are opponents to them. The position, as well as the condition of the muscles, may be imagined by that of a person in the act of attempting to throw a summerset backwards. While in this situation, the patient is desired to take deep inspirations. I direct manipulation, and afterwards percussion, to be employed for one or two hours during the day, gradually increasing them in force according to the influence produced on the patient.

In addition to these means, I usually desire the patient to suspend the body by the arms, and similar modes of exercise, with a view to promote the full action of the pectorales, serrati magni, and postici muscles, &c. on the ribs, to produce the greatest possible extent of elevation of the ribs and sternum, and consequent expansion of the chest.

The benefit to be derived from this plan will, of necessity, depend much on the age of the patient; if the sternum and cartilages have not yet become completely ossified, although the disease may have existed for a considerable length of time, a greater degree of benefit may be expected by a steady perseverance in the means recommended, than if the individual be at an age when the bones have acquired their solid state; and even in the latter case, much may be done, by the increase of muscular power, for the relief of the patient."

In a chapter of miscellaneous observations, Mr. Ward attributes a good deal of certainty to muscular exercise. It is, literally, his primum *mobile.* For example. The state of spasm, says he, in which the sterno-cleido-mastoid muscle is sometimes found, denominated wry-neck, usually has its source in disorder of the stomach and bowels, and, in general, yields readily to purgatives, anodynes, and fomentations. But if the disease should have become permanent, it admits of a cure by the means above-mentioned.

A young lady, between eight and nine years of age, had been troubled a considerable time with spasmodic twitchings of the muscles of the face and head; various remedies had been used, purgatives, mercury, arsenic, without success. Believing that the stimulus of volition might be beneficially employed, she was directed to carry a light piece of wood standing upright on the head, thus rendering it more difficult to balance; and by perseverance in its use, after some time, the disorder was entirely removed. This, no doubt, was an instance of nervous disorder, and such are curable and properly enough treated by muscular exertion. But the wry-neck that depends on chronic inflammation of the muscle or its sheath is undoubtedly not so manageable.

Then, again, the curved legs of rickets have been made straight by exercise.

" The effect of well-directed exercise, persevered in for a considerable length of time, was also exemplified in the case of a young lady, ætat. 11, who had the bones of the thigh and leg curved forwards, in consequence of rickets, at a very early period of life. I had seen the general health suffer much from the use of instruments, and determined therefore to try the effect of exercise only. She was directed to raise herself, and support the weight of the body on the phalanges of the foot, as often and as long at a time as she could every day till fatigue was induced; repeated twice in the day. This was continued between three and four years; at the expiration of that time, the curvature of the bones had disappeared. In this case occasional aperients, and cold bathing during the summer season, were also resorted to."

And club-foot may be managed with equal success by the same means.

*Retardation of the Pulse by Exercise.*—" The influence of exercise in diminishing the frequency of the pulse, is not undeserving of notice in this place. In the case of a young gentleman, whom I directed to use considerable muscular exertion, the first effect was to produce a considerable increased quickness of the pulse; at the expiration of a quarter or half an hour, however, when the immediate acceleration from exercise had abated, the number of beats had been reduced twenty and thirty in a minute. The same effect I have also frequently witnessed in adult age. In a gentleman of forty years of age, whose pulse had regularly, during two years, beat ninety strokes in a minute, it fell to eighty, and subsequently to seventy-five, on using daily strong muscular exercise."

*In gouty concretions of the joints,* excitement of the muscles, whether by voluntary exercise, or other modes, as those of friction, shampooing, or percussion, or a combination of all of them, may be employed with success, observing the caution before given to bring into action the muscles which move the affected joints, and to limit the friction, &c. to those parts only, rather than apply it to the seat of disease.

Mr. Ward recommends muscular exercise in the treatment of stammering, and no doubt the proper use of proper muscles is of service. He concludes with a remark which we quote—some, he says, who have not hitherto directed their attention to muscular exercise as a remedial agent, may perhaps think it has been recommended too indiscriminately in disorders apparently so dissimilar in their nature. It has however this advantage, that provided what may be termed the dose be properly regulated and apportioned to the strength and capability of the patient, no injurious consequences will ensue from its use.

And no doubt there is truth in this, though candour must own that Mr. Ward has his hobby.

THE SURGICAL ANATOMY OF INGUINAL HERNIÆ, THE TESTIS AND ITS COVERINGS. By *Thomas Morton*, one of the Demonstrators of Anatomy in University College, London; and formerly House Surgeon to the Hospital of the same College. Illustrated with Lithographic Plates and Wood Engravings. London. Taylor and Walton. 1841.

Mr. Morton is favourably known to the anatomical world by his previous publications on the groin and perineum. The present work is a worthy successor to those, and will prove, we do not doubt, as great a favourite with students. The descriptions are drawn up with care, accuracy, and conciseness—the plates are lucid and illustrate the text agreeably and usefully—and the price at which the work has been got up is as moderate as could be wished.

---

A TREATISE ON STRICTURE OF THE URETHRA, CONTAINING AN ACCOUNT OF IMPROVED METHODS OF TREATMENT; WITH AN APPENDIX, ON DILATATION BY FLUID PRESSURE IN THE TREATMENT OF URINARY CALCULUS AND OTHER DISEASES. By *James Arnott*, M.D., late Superintending Surgeon in the Honorable East India Company's Service. Second Edition. London, Sherwood, Gilbert, and Piper. 1840.

This is a second edition of an ingenious work. We shall not go into it, but merely pick out the gist of Mr. Arnott's views—his mode of dilating strictures of the urethra.

" A tube of oiled silk, lined with the thin gut of some small animal to make it air-tight, and attached to the extremity of a small canula, by which it is distended with air or water from a bag or syringe at the outer end, with a stop-cock or valve, to keep the air in when received, is a description of the apparatus.

The thinnest silk ribbon of different breadths, with the edges neatly sewed together, so as to make it tubular, and then varnished with prepared linseed oil, which dries upon it, and leaves the surface perfectly smooth and soft, is what I have found to answer best. The gut of any small animal will form the lining; but that of the cat is preferable on account of its thinness and strength. The canula may be of elastic gum, or of the flexible metal used to make the metallic bougies, or of silver; and the injected air may be retained, either by a stop-cock at the outer extremity of the canula, or by a valve at the silk tube or bag itself. This last method is the only one applicable to the insulated dilator, which is a short length or bulb of the lined silk, to be distended and left in the canal, and having a bit of canula in the centre, for the free passage of the urine; the patient while wearing it being little incommoded by its presence.

The dilator, when empty, is introduced or withdrawn with the greatest ease to the patient.

As the shape of the silk tube is in our power, it may be made so as to have any desired form when distended; and the sizes of dilators may be ascertained with as much precision as of metallic bougies. It has the important property also of being permanent in its dimensions. On one or two occasions, where a silk tube of the size wanted was not ready, I substituted sewed bladder; but I found that in the moisture and warmth of the urethra, this yielded in the course of a little time, so as to become of double the original diameter, thus stretching the sound canal on each side of the stricture beyond what it could bear: and in any case where strong pressure were required, such a tube would burst before the effect could be produced.

It possesses strength to bear any degree of pressure which can be useful, for the membrane of the urethra itself would be torn, before a strong silk tube would give way: it is almost needless to add, that by injecting more air, the pressure in any given case is increased, or that it is diminished by opening the cock or valve."

" The manner of using the dilator is as follows: Though in general it passes as easily down to the stricture as a small bougie, yet, on some occasions, especially in irritable urethræ, unaccustomed to the presence of instruments, I have preferred introducing it through a smooth canula, in the manner already mentioned. As soon as the bag is sufficiently within the stricture or strictures, (if more than one exist in the canal,) as much air is to be injected into it as the patient can easily bear; and during the time it remains in the urethra, the future admission or escape of the air is regulated by his sensations; that is, if the feeling of distension abate, more may be injected; but if it should increase into pain, a little of the air may be allowed to escape."

In an Appendix Dr. Arnott thus notices the application of fluid pressure to the surfaces of the body.

" Pressure applied to the surface by bandages, is the principal remedial means in a great many diseases, as chronic inflammations, glandular swellings, diseases of the joints, ulcers, dropsies of various kinds, &c.; and operates by supporting the weakened vessels, promoting absorption, and otherwise influencing the vitality of the diseased structure. But to many parts of the body bandages are applied with so much difficulty as to be a serious impediment to their use; with every care, equable pressure can scarcely be made or maintained by them, and from the difficulty of increasing or diminishing the pressure, which is consequently rarely done in the intervals of the Surgeon's visits, much suffering or mischief often arises.

For the bandage, the equable pressure of elastic air may be substituted with great advantage, by enclosing the part to be subjected to it in a close *double* case of india-rubber cloth, of rather larger dimensions than the part, in order that, upon being inflated, a thin stratum of compressed air may surround it. The outer side of this case, or wherever it is not supported by the surface of the body, must be of strong material to resist the requisite degree of pressure, but the side in contact with the skin should be thin, and of larger dimensions than the other, for the purpose of its coming closely in contact with all the inequalities of the surface.

This case, or air bandage, may have its edges kept in contact by buckles or other convenient means; a double stocking, or long glove may be substituted in affections of the extremities. The inflation can be made by a syringe, or by the contrivance used for filling air beds and pillows.

Pressure by condensed air has already been employed in a few of the affections above enumerated, but so imperfectly, from its being greater at the margin than elsewhere, and thus forming a sort of ligature, as to counterbalance its advantages, or even to be positively injurious. The mode now proposed, enables us to apply a perfectly equable pressure to the most irregular surface, and to moderate or increase it with ease, as the sensations of the patient or other circumstances may require. Should frequent intermissions in pressure be advantageous, they can thus also be easily obtained. By the substitution of such means for the bandage, pressure may not only prove more advantageous in cases where it has already been in use, but be extended to other analogous affections, in which, on account of the imperfect means of application, it has not hitherto been employed."

### Surgery in Paris.

In our last number we made some extracts from Dr. Markham's "Observations on the Surgical Practice of Paris," and exhibited some of the sayings and doings of the hospital surgeons in that city. It is so important to those gentlemen themselves, to us, to our common profession, and to science, that what is wrong should be blamed, what is remediable remedied, that at the risk of being tasked with nationality and prejudice, we shall not shrink from the office of censors. It is not that we wish to drag mere errors to light—to brand mistakes—to lacerate the feelings of the diffident and nervous—to lash opinions contrary to our own. Let those who will play the part of executioners on imperfections do so:—that is not to our taste, nor is it our custom.

But disregard of experience sanctioned by time and supported by numbers, recklessness of consequences, culpable carelessness, looseness of statement and mendacious assertion, insensibility to human suffering, and a passion for display at the expense of the well-being or the life of others—these are crimes towards society as well as science, and the interests of both demand their punishment. Whether the Parisian surgeons be guilty or not guilty, we shall not decide. Those who have watched them are the witnesses, and their evidence it is which we state at the bar of public opinion. If that evidence be unfavourable, we regret, but would not conceal it—if unjust, the aggrieved have ample means and talents for defence. Nor can they, if candid, object to the publicity or even criticism that would put down offences against good feeling, sound knowledge, and morality.

We proceed with our extracts from the work before us, selecting prominent facts or observations without (for it is impossible) an attempt at connexion or analysis.

1. *M. Velpeau on the practice of publishing Successful Cases.*

The occasion which called forth M. Velpeau's observations on the practice of publishing "successful cases" of operations, was an instance in his own practice of extirpation of one of the superior maxillary bones. This operation, to all appearances, was satisfactory in its results: and two months after its performance might be said to have succeeded perfectly; but M. V. was not satisfied, and did not lose sight of the individual. His suspicions were not unfounded; for, five months from the date of the operation, the disease began to show itself again; and this, which in most instances would have been undoubtedly given to the world as a successful case, proved in reality the very reverse. M. V. warned his auditors against taking for granted these vaunted cases; assuring them that there was no part of medical literature in which less confidence could be placed. He mentioned also, at the moment, several cases of phlebitis, which had been published a few days before in the Gazette des Hôpitaux, as having been successfully treated by M. Lisfranc; showing clearly, from the description itself, that these were in fact no cases of true phlebitis at all, but inflammation of the cellular tissue surrounding the vein, or, what M. V. denominates "external phlebitis."

We fancy this applies to England as well as France.

2. *M. Roux's reprehensible Operations for Malignant Tumors.*

Dr. Markham tells us that he has seen M. Roux remove an encephaloid tumor of the testicle in its last stage of degeneration, extending towards the abdomen along the course of the cord—a tumor larger than two fists—it was an operation of great difficulty, and of great pain and suffering to the patient, but was performed in a masterly manner; the enormous wound left was covered with charpie, and ulceration and suppuration proceeded in their course for a month favourably, when there appeared a small tumor rising up in the centre of the wound, as if proceeding from the abdomen; it increased rapidly, and when it had gained the size of an egg, M. Roux again ordered the patient into the operating theatre, and again removed a tumor: a third was not long in showing itself, and M. Roux, at last deeming all attempts to remove an immovable disease useless, sent the man into the country to die.

Surely this is unworthy of the knowledge and humanity of the day. It is just what occurred in this country many years ago, before the nature and properties of the malignant morbid growths had been properly investigated or could be understood. We need not add, that such practice would not be tolerated in an English hospital now. What surgeon in this country would amputate under these circumstances?

A man presented that well-known aspect which is an infallible indicator of the progress of cancer; the thigh was enormously swelled, and covered with blue veins; a small part of the highest part of the thigh appeared unaffected by the disease, and here M. Roux amputated the limb: the operation was most difficult and prolonged; a very great quantity of venous blood was lost; the man fainted again and again after the operation, and then rallied, but he never recovered from the shock; his face was sunken and pallid and mournful, his pulse quivering, and agitation extreme, and he died on the third day after the operation. The fungoid disease proceeded from the centre of the bone, of which it had completely altered the texture; the muscles, also, all round the thigh could not be distinguished from the diseased mass.

3. *Loss of Sensibility from Injury of the Infra-Orbital Nerve.*

The man who was the subject of this injury, had fallen, and in falling had struck the lower part of the orbital ring violently against some projecting object; this blow broke in, and caused a depression of the bone above the infra-orbital canal, and thence pressure on the infra-orbital nerve, and the consequence was complete loss of sensation in all the parts which it supplies with filaments.

4. *Extirpation of the Fifth Metacarpal Bone. Inflammation of the Carpal Joints. Advantages of Extirpation of the First Metatarsal Bone.*

M. Blandin performed this operation on the hand of a young man: the bone was affected by caries, was much enlarged, and had produced several fistulous openings in its vicinity; in this operation, which M. Blandin performed by a simple incision carried along the inner side of the hand, the synovial membrane, which is continuous through the bones of the carpus, must be opened, and the consequence in this case was violent inflammation of the whole of the hand, suppuration took place beneath the fascia, and numerous openings were made in the hand to give exit to the pus—after a time the inflammation subsided, but the lower part of the wound, whence the bone was taken, did not heal. Three months after the operation, this fistulous opening still existed, and gave exit to a glairy discharge; the little finger was quite motionless, and the patient did not seem to have the slightest power over it, so that probably some of the other bones were affected in this scrofulous subject; what motion the finger might have regained, if the operation had been successful, it is difficult to say, but from analogous ablations of bone, as of the jaw, &c. it is well known that deposits of a cartilaginous nature supply well the deficiency of the bone which is lost—the inflammation consequent on this operation is well worthy of remark, and in respect to this character it may be compared to the following operation of extirpation of the first metatarsal bone of the foot where a different arrangement holds as to the synovial membrane. This operation M. Blandin performed for caries and enlargement of the bone; the consequent inflammation here was much less than in the last case, and the reason may no doubt be because only one joint is opened. This operation is neither difficult nor dangerous, and is undoubtedly much preferable to taking away the whole of the great toe, for when this is done, it happens, that the foot may turn over, from want of its natural support in the ball of the great toe. M. B. observed that it was much better to take away the whole of the bone, than only a part, for in the latter instance, a troublesome caries often ensues, and, as had happened to himself, may require a second operation. As the articulation of the first metatarsal bone, with the internal cuneiform bone, is situated at exactly the middle point, between the heel and the extremity of the great toe; an incision is made from this point along the inner border of the foot, to the first phalanx of the great toe, and a transverse incision is made at the tarsal extremity of this incision. The tarsal, *or greater* extremity of the bone is first dissected out, and then the operation is easily completed. The anastomotic artery passing between the two first metatarsal bones, is almost always opened, and was in this instance. M. B. took one branch up on the dorsum of the foot. The result of this operation I cannot give, as when I left Paris, four months after the operation, the subject of it was still in bed with a fistulous opening in the wound."

5. *Wound of the Fore-arm—Diffuse Inflammation of the Deep Cellular Membrane —Propriety of lengthened Searching for Arteries?*

A healthy man, young and vigorous, was brought into the Hôtel Dieu, with a lacerated wound of the lower and front part of the fore-arm. He had, while in a state of inebriety, struck his arm down upon a glass, and broken it in pieces —several of the fragments were sticking in the soft parts. The depth of the wound, which was contused and lacerated, was not clear; from its situation the ulnar nerve was probably divided; and from the great loss of arterial blood, which had taken place, probably the ulnar artery also; compression, above and below the wound, in the course of the ulnar artery, M. Blandin thought very unadvisable; and, although the bleeding had ceased, he sought for the extremities of the divided artery, and tied them—an operation which required much dissection and labour, from the laceration of the surrounding tissues; the wound was then dressed with straps and bandage. The next day there had been no bleeding, and the wound appeared well. On the third day one of the ligatures came away; but there was swelling above the wound, and pain, so the plaister straps were removed. On the fourth day the swelling was increased, and there was every appearance of violent inflammation beneath the aponeurosis of the fore-arm; it was swelled in all directions, presented an unusual roundness, and that most remarkably in front; the skin was tense and *colourless,* pressure gave a sensation of deep tension, and obscure fluctuation, œdema of the cellular tissue, distention of the superficial veins (from the pressure on the deep veins of the fore-arm)—a kind of insensibility in the limb. One hundred leeches were ordered to be applied to the fore-arm, and a low diet enjoined. On the fifth day there was the same swelling, more insensibility, heat hardly natural, much fever, and the leeches had not at all relieved the symptoms, and there was every reason to believe that gangrene had already commenced; three long incisions were made through the aponeurosis, in the length of the fore-arm. On the sixth day the skin had a blackish tint, and gangrene had evidently seized the fore-arm and hand; to prevent its extension three incisions were made in the arm, and camphorated applications were ordered to be applied. The low fever was much increased, the pulse weak and quick, and delirium had occurred during the night, the belly tympanitic, and general prostration very great. Cold applications were ordered to the head, and slightly stimulating medicines to be taken. During the course of this day all the symptoms increased, and the patient died.

We make no question whatever that this was essentially a case of diffuse inflammation and suppuration in the intermuscular cellular membrane. We doubt if any thing, save, possibly, early amputation, could have saved the man. But most assuredly a *hundred* leeches would not do so. Such practice is of very questionable propriety, in diffuse inflammation of cellular tissue it is probably highly injurious. Such local depletions must sink the powers of the part and of the system, and tend to prevent the formation of that barrier of lymph which is the only chance of safety that nature has to offer.

Dr. Markham asks if the lengthened search for the ulnar artery was right. This question seems to him answered by the case of a patient of M. Velpeau, a man, who in the same week, (during the height of the carnival,) met with a very similar accident. This man, also in a state of drunkenness, struck his arm down on a glass, broke this, and opened the radial artery, just above the wrist. M. V. merely applied pressure, and the wound healed without an accident.

Dr. Markham refers to another case of interference, which he deems, with justice, injudicious. A man fell down some steps on a bottle, broke it and wounded (with a deep clean cut) the parts above the wrist, across the course of the ulnar artery. A great quantity of arterial blood followed at the moment; he was however immediately dressed by a surgeon, with compresses and bandages, and sent to the Hôtel Dieu. M. Blandin saw him twenty-four hours after the accident; the bleeding had quite ceased, and the arm was quite easy. M. B. removed the bandages, &c. enlarged the wound, and after half an hour's search, (rendered difficult by the tendon of the flexor carpi ulnaris having been partially divided by the glass,) the ulnar artery was found untouched. Of course the operation did nothing, but inform the surgeon that the artery was sound, except causing much pain to the patient, and a probability of subsequent inflammation.

Dr. Markham thinks that in wounds of the ulnar and radial arteries *near the wrist*, compression, well applied, is of itself sufficient to arrest the hæmorrhage, and that the search for the wounded extremities of the divided artery, (generally rendered difficult by the laceration of the soft parts around, caused by the injury,) is unnecessary, productive of much pain, and very liable to be the cause of violent inflammation.

If the vessels cannot be reached with facility, we agree with Dr. Markham in considering it better to trust to careful compression. We have ourselves had a case of wound of the ulnar artery near the wrist with a knife, one of the radial where it dips between the heads of the abductor indicis, and one of the anterior tibial, where it dives between the metatarsal bones, all of which did perfectly well with compression. Of course if the ends of the wounded vessel present themselves, or can be tied with little disturbance to the parts, that would be the better plan to adopt.

### 6. *Deceptive and Fatal Wound of the Chest.*

A married woman entered the Hôtel Dieu, on account of a wound in her back from a knife. She said, (as it turned out falsely, for she had been purposely stabbed) that her husband had accidentally struck her with a knife, which was *broad* at the point.

On examining the seat of injury, an incised wound was discovered at the internal and lower border of the scapula, about an inch long, running in the direction of the length of the ribs, between two intercostal spaces, just external to the angles of the ribs. There was much effusion of blood round the edges of the wound, the lungs appeared perfectly intact, the vesicular murmur was clear, and no crepitation could be distinguished. There was no embarrassment of the respiration, no spitting of blood: hence no sign of injury of lungs, or effusion of blood or escape of air into the pleura; so that this could scarcely be considered otherwise than as a simple incised wound; in fact, such was the distinct diagnosis of the case. The length of the wound was to be accounted for by the breadth of the point of the knife. The case was held as of the slightest importance; but still, as M. B. thought on the bare possibility of a deeper injury, he ordered blood to be taken from the arm.

On the second day the wound was closed, and everything was perfectly well; on the third, she thought of leaving the hospital, as her cure seemed complete; she, however, for some cause, did not obtain her dismissal. On the seventh day (three days ago,) her case suddenly presented quite a different aspect; her respiration was difficult, quick, and jerking; pain in the side on pressure; suppuration in the wound, and the discharge mixed with the coagula of the ecchymosed blood; a friction sound was heard in the chest. She was immediately ordered to be bled, and thirty leeches were applied over the seat of pain, then a poultice, and the strictest diet enjoined. The next day she was more depressed; the chest was dull to percussion at the lower part, and respiration inaudible below; but at the upper part of the lung œgophony was distinctly recognized. She was very feeble, and her intellects were confused; a large blister was applied over the chest. Yesterday she was more enfeebled; a yellow tint of the skin and conjunctiva, intimated some affection of the liver, whether arising from continuous inflammation or secondarily. To-day the dullness is increased over the chest, and the respiration is more difficult; the effusion of pus into the cavity of the pleura was very evident; it flowed out in abundance from the external wound mixed with flocculi. She died on the following day.

On examination of the chest, the pleura was found (on the side where the wound existed,) highly inflamed, and a great quantity of pus existed in its cavity; the lung was compressed to the back part of the chest, and a wound existed in the pleura, corresponding to that on the outer side of the chest.

This case is certainly calculated to inspire caution. It would seem that, after the first, the attention directed to it was insufficient, and it was only when grave symptoms arose that *that* was re-awakened. All wounds of the parietes of the great cavities should be watched.

7. *Difficulty of Matter escaping through Muscles.*

"As to the difficulty of liquids passing through openings in muscular fibres, *i. e.* openings parallel to their length, I might mention a case where M. Lisfranc and another surgeon, distinctly determined the presence of pus under the pectoral muscle, an incision was made through this muscle, but not a drop of pus, or fluid of any kind, followed. Both the surgeons were amazed: 'Donnez-nous une prise de tabac,' said M. L.; and then suddenly the thought struck him, to separate the lips of the wound, on doing which, a jet of pus immediately sprung out."

8. *The Politest People in the World.*

M. Lisfranc, remarking on a doubtful case of a tumor on the metacarpal bone of the thumb, said, it was a pity it had not fallen into the hands of some villainous perruquier, he would have made a fine lesson of it, running through every thing, *prepared* upon the subject. Il faut le couper, continued M. L., to find out its nature, and what does it signify if it is lamb or mutton, beef or veal, the only practical point is its extent and relations.

To understand the force and the good taste of this, our readers should be apprised that the "villainous perruquier" means every other hospital surgeon of Paris. Can we wonder that the profession holds a low place in public estimation in France?

9. *Rough Surgery.*

A woman, æt. 64, of hale and healthy appearance, came into the Hôtel Dieu, under M. Blandin's charge. She journeyed to Paris, from the country, under the advice of the surgeon who attended her there, to undergo amputation of the leg, for this tumor, which he deemed of a cancerous nature.

She says that about three or four years ago, she, for the first time, noticed a small swelling below the internal malleolus, a tumor, of whose origin she could give no account, except that it might have been caused by the pressure of the heavy wooden shoes worn in the country. She then experienced no pain or inconvenience from it, and she took no further notice of it; but it has gone on gradually and slowly increasing up to the present moment. It is situated immediately below the internal malleolus of the left foot, is round, a little larger than an egg, slightly flattened above; it moves with the motion of the foot, and seems to have no connexion with the ankle-joint, or the tibia; its base is hard, like an exostosis, and it has a hard fluctuation in the centre—is elastic; the skin surrounding it is of a violet hue, and varicosed branches of the internal saphena vein course around it. The disease is quite local; neither the foot nor the leg is affected by it, motion is quite free, and the lymphatics and ganglions healthy. The woman's general health is good: but she complained that her sleep was latterly somewhat broken by starting pains in her toes (which might be supposed to depend on the pressure of the tumor on the plantar nerves.)

M. Blandin thought it was a tumor arising from inflammation of the periosteum, which had been probably bruised, or in some way injured, the base having become hard, from the deposition of osseous and cartilaginous matters. As it seemed attached only to the astragalus and calcaneum, M. B. determined to make a crucial incision over it, dissect back the flaps, and attempt its removal from its connexions; if this was impossible, or if it turned out to be of a fungoid character, and projecting deeply among the muscles, nerves, &c. of the foot, M. B. decided, that he would immediately amputate the leg.

The operation of dissecting this tumor was tedious, and appeared to give very much pain to the patient; when it was completed the tumor was ascertained to be situated in the body of the posterior tibial nerve, and was a cartilaginous deposit among the filaments of this nerve, developing itself in a direction towards the foot, and spreading among the nervous fibrillæ of the internal and external plantar nerves. M. B. having determined that it was impossible to remove the tumor, at once amputated the leg, at the junction of the middle with the inferior third of the limb, and by the circular operation.

The day after these severe operations, the patient was in a high state of traumatic fever, and her nervous system seemed much agitated. On the second day, the stump looked well, and she appeared more tranquil, and the fever was certainly diminished. On the third day she was worse, her face was very expressive of suffering and dejection. She had been delirious during the night; her pulse quick and feeble, tongue dry and brown, the stump unhealthy in appearance, and no sign of vigorous action in it; her respiration was hurried, and she died during the day.

There can be no doubt that this patient died from the harshness of the shock upon her nervous system.

### 10. *Operations for Hæmatocele.*

M. Velpeau rejects M. Roux's* practice of extirpation of the testicle, and Dupuytren's, of taking away only a part of the tunica vaginalis. His method is either to evacuate the sac, if its contents are fluid enough, by means of the trochar, and then to throw into it an injection of iodine. If the cyst contains coagulated blood, &c. then he makes an incision into it, large enough to give free exit to its concreted contents: M. V. avers that this operation succeeds perfectly.

### 11. *Hernia Humoralis—Epididymitis.*

"This disease is by many authors considered as an inflammation of the body

---

* "I am not certain if this was not Boyer's operation."

of the testicle. One of the latest English writers says, ' the body of the testicle swells, with great pain and tenderness.' M. Blandin, is of opinion, that in ninety-nine cases out of a hundred, the epididymis* and vas deferens are the seat of this inflammation, and not the testicle. Several cases, which he pointed out in the Hôtel Dieu, and which I observed in other hospitals, strongly confirmed this view, which is strengthened by anatomical considerations. It is difficult to conceive, how so dense coverings as those which surround the testicle, could admit the rapid swelling which so often takes place in this disease; and if the progress of the malady is carefully watched from its onset, it is not difficult to determine that this swelling is really seated in the epididymis and vas deferens. The epididymis, when much inflamed, spreads out, swells, and embraces the whole of the testicle, so that when the inflammation is at its height, this cannot be felt. But before it has reached this point, it often happens, that in some part of its circumference, the testicle may be felt of its natural size and hardness. M. B. pointed out this circumstance several times, and it was clearly appreciable, the hard body of the testicle being felt, where it was as yet uncovered by the swelling epididymis. In all the cases (and they were a great many) which I observed, there was not the slightest necessity of calling in the aid of sympathy to account for this swelling; for the sensations of the patient could follow the inflammation proceeding along the course of the cord."

No doubt of all this.

12. *The Starch Bandage.*

"The treatment of nearly all kinds of fractures by the starch bandage, is now almost generally adopted by the Parisian surgeons: M. Lisfranc and M. Jobert (of St. Louis) alone, I believe, raise their voice against the practice. It is adopted in the practice of Velpeau, Roux, and Blandin, and with the most favourable results. The invariable practice of all the surgeons who employ this method, is, to dress the fractures for the first days merely with the common splints and bandages; but when the moment of inflammation has passed, and when the process of union may be supposed to have commenced, and absolute rest rendered requisite, to apply the immoveable apparatus."

M. Roux treats all his fractures during the first interval by a linseed-meal poultice, smoothly applied over the fracture; then bandages, pads, and splints: these are changed every day, until the starch bandage is applied. This meal poultice is sometimes, but not always, employed by M. Blandin. Dr. Markham does not know how these gentlemen manage with fractures of the thigh.

13. *Complete and Incomplete Fractures of the Clavicle.*

In infants, said M. Blandin, in whom the periosteum is very strong, this is often not ruptured, and then there is no displacement. This, sometimes, though rarely, happens in adults, and has been often the cause of error and mistakes. M. B. said, that he was the first who noticed this species of fracture. There is no displacement when the fracture is between the coraco-clavicular ligament, and the acromio-clavicular articulation, so that this displacement is easy in some cases, impossible in some, and rare in others. M. B. had treated a case of fracture without rupture of the periosteum, and consequent displacement in an infant at the Hôpital Beaujon. There is no difficulty in recognizing this fracture, if its possible existence be only kept in view. Dupuytren almost entirely abandoned the treatment of this fracture to nature, merely placing the arm

---

* Engorgement of the epididymis may be always destroyed, M. Lisfranc observed, by leeches along the cord. It seldom degenerates into scirrhus, but rests a long time in the same state. There are, however, exceptions; where it does, therefore, always combat these engorgements.

of the individual in a sling; but he was decidedly wrong. The pieces ride on each other, and hence irritation and inflammation of the periosteum, great pain, and consequent deformity. Dessault has pointed out the very best treatment of this fracture; has shewn the necessity of continued extension, of raising the arm, and separating the fragments, by placing a cushion in the axilla, of pressing forwards and inwards over the chest, and of bandaging the whole arm; the great object of all being to force the shoulder backwards and outwards. M. B. followed this treatment in two cases, applying the starched bandage to fix the arm, and the results exceeded any thing Dr. Markham had seen in England.

We would observe that many surgeons have noticed the incomplete fracture of the clavicle, as of other bones, which takes place in the young. In these cases, however, the bone breaks like a green stick, and it is not the periosteum only that is untorn. We would add our testimony, such as it is, to the superiority of Dessault's plan of treating fracture of the clavicle.

14. *How are we to Operate for Phymosis?*

M. Ricord always performs the operation of circumcision in this complaint, when it is possible, but when the phymosis is complete, when the prepuce is small, and tightly applied to the gland, then another operation must be performed. In performing this, M. R. is not contented with the common incision, carried along from the opening of prepuce to the base of the glans, (on the upper part of the penis;) but after making this, he practises another in a parallel direction, on the lower part, at the frænum; then seizes the flap left on either side with a pair of forceps, and cuts them off with the scissors. This operation is long, comparatively, and painful; but M. R. declares that the result, which is really excellent, well repays the extra pain, for scarcely any deformity ensues; and this is of the highest possible importance, added M. R. for there is no part of the body where surgery ought to be more coquettish.

In performing this operation (and the same rule applies in all other operations on the penis) the points of the incision should be marked out by nitrate of silver, at the spot where the knife's point should make its appearance at the base of the glans. In circumcision, also, the line of incision should be marked; a narrow, fine, sharp knife should be used. After making these observations, M. R. proceeded to operate on a case of the nature mentioned above, where the prepuce was firmly applied to the glans, and was very short. M. R. unwittingly made use of a very large, dull knife, and did not mark out the point where the incision ought to terminate. The consequence was that the knife, when pushed forwards, instead of piercing the skin at the base of the glans, puckered up the skin before it, so that when the knife was forced through, and the incision completed, the skin of the dorsum of the penis was ripped up very nearly to the pubes.

M. R. said that he had seen Dupuytren and his own predecessor at the Hôpital du Midi, cut off the glans penis as well as the prepuce, in operating for phymosis by circumcision. M. Blandin's operation, and M. Velpeau's also, consists merely in one single incision along the dorsum of the glans, through the prepuce.

We have ourselves, when the prepuce has been very long, performed an operation similar to M. Ricord's. We were not aware that it was done by any but ourselves. In most cases, however, it is unnecessary, simple division of the dorsum of the prepuce sufficing. If there is no part of the body where surgery should be more "coquettish," there are few where pain is less liked, and the majority of patients think one cut enough. By-the-bye, a good illustration that of French surgery, when M. Ricord having said that a sharp knife and a caustic mark were indispensable, took a blunt one, and made no mark at all! On the advantage of the former we quite agree with him; the *necessity* for the latter is not so obvious, if a surgeon takes care to manage the outer fold of foreskin with his thumb, so that he shall transfix it at the proper place.

15. *Death from an Autoplastic Operation for Fistula Urethræ.*

The following case is not uninstructive, nor is it, perhaps, without its warning lesson.

The patient was a healthy man, about 45 years old. The disease had existed from infancy, and no remedy had been tried for its cure, when he presented himself at the Hôtel Dieu. The fistulous opening was situated in the median line in front of the scrotum, it appeared very small when the penis was retracted, but much larger when it was extended, being then nearly half an inch in length; there was no doubt as to its being an urethral fistula, for a probe could be passed directly into the urethra. On being more narrowly questioned, he at last admitted that when a child he had put a ring round his penis, and this was no doubt the origin of the fistula, and of the destruction of the urethra at the part, for M. Blandin, in introducing a bougie observed, that the lower part of the urethra was entirely destroyed. M. B. determined to attempt its cure by Earl's method of operating, by an autoplastic operation—for this purpose a portion of the integument above (nearer the root of the penis) the fistulous opening, about eight lines square, was entirely dissected off—the callosity around the opening was also removed, and then a flap half an inch long was dissected up from *below* the fistula, and was then drawn over it backwards towards the root of the penis, (thus making the penis, as it were, describe a curve with the concavity below) and was united by sutures to the broad flat surface from which the skin had been already removed. M. B. hoped, by this means, to prevent any small quantity of urine, which might pass from the urethra, being a hindrance to adhesion in every part of the surfaces brought together—an elastic catheter was passed into the bladder. The second day after the operation the penis and the parts around the wound were enormously swollen, slight union appeared to have taken place in one point, the urine passed freely by the catheter, and the patient had noticed none passing by the wound ; one or two of the pins (by which the sutures were formed) were removed, by reason of the great constriction they caused; the next day (third) no further union seemed to have taken place, and all the sutures were removed. After this the patient (who was a most irritable subject, and who never ceased lamenting the day he applied at and entered the hospital) went through a series of misfortunes, which terminated in death; high inflammation arose in the parts, no more union took place, and that which had taken place was destroyed; pus, blood, and urine, began to flow from the wound; the man became very restless and feverish; two or three abscesses formed around, incisions were made to evacuate them: the inflammation appeared to have destroyed part of the urethra, for after eighteen or nineteen days from the operation, infiltration of urine took place, and death was the consequence.

16. *Dr. Markham a warm Patron of the Speculum.*

" The virulent abuse which the use of the speculum vaginæ has excited, as being immodest and unnecessary, can only be sanctioned by those who are ignorant of its purport, and have never seen its employment. Those who have witnessed its assistance—(I might say, the absolute necessity of its use, for it appears to me, to be to the vagina and uterus, what the stethoscope is to the lungs)—cannot for one moment hesitate to affirm, that humanity must gain infinitely by its aid. An unbiassed individual who has once seen its proper application, cannot but be struck by its utility; and this once proved, of course the idea of immodesty ceases at once.

To what does the English physician attribute those acute pains which females suffer in the middle and early periods of life? the violent pains felt in the hypogastric and lumbar regions, and in the nates, at the back of the sacrum, in the groins during walking,—and these pains, accompanied by an abundant discharge, of a white colour? The physician can give no cause. The very name itself applied to these complaints, the *fluor albus*, shows plainly how the effect has been taken for the cause; and the futility of the treatment employed, the endless round of injections and tonics prescribed, might be enough at least to give a suspicion that the true nature of the disease had yet to be described."

Now *we* plead guilty to "abuse" of the speculum—*we* have said that its employment as a common means of diagnosis is immodest—*we* have protested that, in the majority of cases, it is unnecessary. And, under Dr. Markham's fire, we do not flinch from repeating this, nay more, we assure him, that should he endeavour to carry into practice in this country Continental notions of what is *decent*, he will assuredly make shipwreck of his character and fortunes, as some of our travelled youth have done. We do not mean to rip up an argument only just skinned over, and as sore as recent, nor to defend the opinions we have expressed—opinions not founded on any affectation of boisterous prudery, but the simple utterance of those sentiments of delicacy which honourably characterise English society. We *do not* say that the speculum should be proscribed—but we *do* say that it is used to an excessive, and a disgusting extent in the Parisian hospitals. If we wanted evidence of this, we should find it in Dr. Markham's own queries and statements. As if English physicians were so ignorant as to know nothing of the general symptoms of congestive and inflammatory affections of the uterus,—as if "the touch" could give no useful indications—as if, in all cases, the filthy speculum must be introduced—as if the following opinions founded on its constant employment were not proof of the fallacy of the results to which such employment leads!—

"*Chlorosis* is caused, some say, by alteration of the blood; and I do not deny, says M. L., that cases of this nature may exist, and that there may be some therapeutical agents capable of inducing an alteration in this bad constitution, and may cure the disease; but it is also, most undoubtedly, caused, in some instances, by engorgement of the uterus, and then are these preparations perfectly useless. And is the physiology of the disease so difficult as to be incomprehensible? When a new function first takes on its office, is it always subject to disturbance? and to this disturbance is not the uterus subject at puberty? Is not this the pure induction, spite of the books of the perruquiers? These engorgements are to be known by the low sinking pains in the lower part of the abdomen, pains in the loins, back, groin, &c. and above all, are to be felt either by the rectum, or the vagina, or by both. M. L.'s treatment is entirely antiplogistic, and consists in local and general bleedings most especially."

We fancy that the cases of chlorosis which would be benefitted by local and general bleeding must be few indeed. But we quit the subject.

### 17. *Amputations in Paris.*

*Circular Amputation the Favourite.*—"I never once saw the flap operation performed in Paris: the circular appears the only one recognized by the Parisian surgeons. This steadfast adherence to the ancient method, I have no means of explaining, as I never heard mention made of the flap operation, even as a subject of discussion."

Then surely M. Lisfranc must have turned round. By the way, this is an answer to those who are always telling us that it is only a few bigotted surgeons in England who still stick to the circular operation. Paris has been pointed to, as the city of flaps. Tout au contraire—not a flap is to be seen there! But that there should be no discussion, no "palaver," as the negro consultants have it, on the subject, is queer. Malgaigne's little book, last published on these matters, would not lead us to expect this. After all, we suspect that the results of the circular operation are the best.

18. *Want of Success after Amputation.*

" As regards the healing of the stump, it is almost impossible to make a comparison between the results of the flap operation, now so generally practised in England, and the results of the circular, as performed at Paris,—for several of the leading surgeons still hold it, both by precept and example, as highly unscientific, to attempt union by the first intention, and their reason is, because this first intention so very often fails ;—but why does it fail? No man in *England* can guess ; but I think, that if any unbiassed individual will pay a visit to any of the large Parisian hospitals,—follow the changes in the wound of an individual, who has just entered for an injury, being previously in perfect health, —if he will notice the characters which almost every wound takes on—the ever existing erysipelas, and its distressing consequences, he will at once allow that there exists some other cause than the idiosyncrasy of French limbs, or the particular nature of the healing process itself, to account for its failure. Moreover, are all the rules of the operation properly attended to? I doubt this. The heat in the hospitals is most oppressive ; ventilation, or opening of a window, is not permitted ; each bed in covered, above, and on all sides, by curtains ; and, what seems almost incredible—certainly incomprehensible—when an individual has undergone an operation, the curtains of his bed are *doubled* on every side, and he is covered up with as much care from all currents of air, as if he were an exotic in the hotbed of a gardener. If the records of the medical annals of the Hôtel Dieu for centuries past could be opened, I do fear, that they would contain a most terrible picture. Every epidemic has raged in this hospital, and always with the most frightful results. To give an instance : in 1746, in the month of February, of twenty women attacked by puerperal peritonitis, in the wards for lying-in women there, scarcely one recovered ; in the years 1774 and 1775 one in every seven women who were attacked, died, and seven out of every twelve who were delivered there were attacked. In one winter Dupuytren lost twenty-one out of twenty-six amputations below the knee. And let any one who wishes to be convinced, visit that hospital at any time, and see erysipelas always present—often raging—in the surgical ward ; and he will have much less difficulty than he had before, in understanding why the wounds from operations almost always fail to heal by the first intention. No doubt, the Hôtel Dieu presents the worst picture of Parisian hospitals ; but the same circumstances prevail, to a greater or less extent, in every hospital. Moreover, as to the dressings to produce union, are they such as are appropriate? M. Blandin (who is the only gentleman who attempts the first union) says, no ; and in fact, accounts for the failure of his *confrères*, through their faultiness on this point. M. Lisfranc never attempts union by the first intention ; and he adds, occasionally, another peculiarity to his operation, viz., slitting down the lower flap, according to the invariable proceeding of Baron Larrey ; and this, with the intention of being enabled, when he pleases, and when granulation has commenced at the bottom of the wound, to bring the parts into more perfect contact. The only possible advantage which this addition seemed to me to give, was its allowing free issue to any puriform matter, and preventing any collection taking place. Its obvious inconvenience, in uselessly enlarging the wound, struck me as quite condemnatory of its practice. M. L. always dresses the stump the day following the operation. M. Roux heals stumps by the established method ; though now and then he attempts the first intention ; but his rule of practice I could not discover. The only good stump I saw in his ward, was one healed by the first intention in fifteen days after the operation. The appearance of a large, red, flat surface, covered with granulations, and often with a bone projecting from the centre, was a curious view to an English eye, and it was one with which I was made quite familiar in M. R.'s wards. The results of his practice, I should say, were unlucky, for I have seen exfoliations of bone take place—abscesses form in all directions—

in fact, I believe, never an amputation (where union by granulation was attempted), without some accident. The reason of the frequency of these secondary accidents may, I believe, be in great part sought in the unhealthiness of the Hôtel Dieu, for they happened also to M. Blandin; but in part also, from the nature and conditions of the cases on which M. Roux operates. Thus, I have seen M. R. amputate an arm below the shoulder, in an individual in the very last stage of hectic and feebleness, and whose whole arm was a mass of suppuration: two days after the amputation, an abscess was opened beneath the *pectoral* muscle, and an enormous quantity of discharge evacuated; the abscess reached in every direction, and the man died on the third day. In the dressing of stumps, M. Roux, as in all other cases of operation, is guided by rule; and this, I believe, prescribes the fifth day after the operation as the day for the first dressing.

M. Blandin always attempts union by the first intention; but he follows a method somewhat peculiar to himself in his after-treatment of the stump—a method which, as he himself affirms, is the cause of the greater success of himself than of his colleagues in the sequelæ of his amputations. It consists in invariably examining the wound the day after the operation, and for the following reasons—that no injury can result from the examination; that, by this, the state of the parts can be exactly determined; that, if necessary, by removing one of the plaister straps, free issue may be given to any discharge that may have accumulated in the wound; and that, if union has taken place in the whole length of the wound externally, (for M. B. asserts, that it is totally impossible, under any circumstances, for union by the first intention to take place in the depth of the wound,) it is necessary to introduce the forceps to break down the adhesions in some part, (the part where the ligatures project,) in order to give free issue to the confined seropurulent discharge, which almost always collects; that the confinement of this discharge will, and very often does, cause great swelling of the stump, pain, abscesses, and total prevention of union by the first intention. These reasonings of M. B. seem founded on true and just grounds, and are well worthy of notice; and, as I said before, want of attention to some of these points, may have been in part, cause of the failure of the first union in the hands of other surgeons at Paris. M. B. considers torsion as effectual in arresting hemorrhage of the arteries, as the ligature, and has successfully employed it on almost every artery that is divided in amputation, but never employs it at present, except occasionally, to demonstrate its effects to those who follow his clinique; and for the reason, that torsion really retards, instead of favouring adhesion by the first intention. In applying torsion, great tearing of the surrounding parts is caused, and consequent inflammation is thus often produced, arising from the difficulty of seizing the vessel itself, and from seizing and twisting more parts than the vessel. In regard to amputation below the knee, M. B. observed, that the operation immediately above the ankle-joint, was much less grave than that in the point of election, and that statistics proved this, (which we can very well imagine beforehand), for in twenty-five amputations immediately below the knee, twenty-one deaths took place, while in fifty cases at the lower third of the leg, six only died. [M. B. did not mention whence these statistics came; but in reading some remarks on Dupuytren's practice, I was struck by the circumstance mentioned in them, viz., that in one unfortunate season, when the Hôtel Dieu was particularly unhealthy, M. D. lost twenty-one out of twenty-five amputations. The statistics given by M. B. seem too preposterous, if we consider the deaths as occurring under ordinary circumstances.] M. B. said, that he fancied he had seen gangrene happen in amputation in the lower third of the leg, from the small quantity of nerves and vessels there."

What do our readers say to this? The faults of French surgery are nowhere more glaring than in the after-treatment of amputated limbs.

THE ANATOMY OF THE NERVES OF THE UTERUS. By *Robert Lee*, M.D. F.R.S. Physician to the British Lying-in Hospital, and Lecturer on Midwifery at St. George's Hospital. London: H. Bailliere, Regent Street. 1841.

OF our worthy and able friend, Dr. Robert Lee, it is not necessary for us to speak in commendation. His unwearied industry and professional enthusiasm have obtained for him an European reputation in pathology—we may now add, in anatomy. Right or wrong, the investigations which have produced the work before us, stamp their author as one destined for philosophical discovery.

Dr. Lee first cites, for the purpose of exhibiting the confusion that obtains in description and opinion, the conflicting statements of authorities respecting the uterine nerves. These we need not repeat—they lead only to the conviction that fresh and more accurate inquiries are necessary—and that, prior to the dissections which our author has made, the records of none were complete or convincing.

Dr. Lee describes the nerves of the gravid uterus in the seventh month—those nerves in the sixth month—from two specimens, in the ninth month—in the fourth month—in the third month—ten days after delivery—the nerves of the unimpregnated uterus—and, finally, the nerves of the uterus in the mare.

The first preparation of the uterine nerves was made by our author in April, 1838. It was deposited in the museum of St. George's Hospital, on the 1st of October of that year. The nerves were of the *Gravid Uterus in the Seventh Month.*

2. *Nerves of the Gravid Uterus in the Sixth Month.*

On the 18th December, 1838, a woman in the sixth month of pregnancy, died in St. George's Hospital, a few hours after the fœtus and its appendages had been expelled. The description of the nerves is exceedingly precise, and we shall introduce it.

"Behind the uterus, the aortic plexus divides into the right and left hypogastric nerves. These nerves soon sub-divide into a number of branches to form the right and left hypogastric plexus. Each of these plexuses, after giving off several branches to the ureter, rectum and uterus, descends to the side of the neck of the uterus and terminates in a large oblong ganglion. The left hypogastric plexus first sends off from its upper and anterior part, some small branches to the ureter. About midway between the aortic plexus and the ganglion at the cervix, the hypogastric plexus sends off several considerable branches directly into the upper part of the cervix uteri, which spread out under the peritoneum of the body of the uterus. The hypogastric plexus then gives off a large branch, which passes between the ureter and uterus, to the trunks of the uterine veins and artery. This branch enlarges and becomes thin and broad as it approaches these vessels, and terminates in a great plexus of nerves, which completely encircles them. This plexus is joined below by several branches, which proceed from the anterior and superior part of the ganglion, and which pass on the outside of the ureter to the plexus, around the vessels. From the inner surface of the ganglion, several branches go to this plexus which run on the inside of the ureter, so that a loop of nerves surrounds the ureter, as well as the uterine artery and vein. From the plexus surrounding the vessels, three large trunks of nerves proceed upwards with the vein to the upper part of the uterus, enlarging as they ascend. The posterior branch sends off in its course smaller branches, which accompany the ramifications of the uterine vein, on the posterior surface of the uterus, and spread out upon the inner surface of the peritoneum. Passing upwards beyond the junction of the spermatic with the uterine vein, and running between the peritoneum and a great plexus, situated on the body of the uterus, it spreads out into a web of thin broad branches and slender filaments, some of which are inserted into the muscular coat and peritoneum, and others follow the veins and arteries to the fundus uteri, and pass with the vessels into the muscular coat of the organ.

The middle and anterior branches closely adhere to the uterine vein as they ascend and form around it several plexuses which invest the vein. From these plexuses, branches are sent off to the anterior surface of the uterus. These nerves ascend and closely unite with the great transverse plexus on the body of the uterus.

This plexus on the left side arises near the mesial line on the back part of the uterus, midway between the fundus and cervix, from a mass of fibres which adhere so firmly both to the peritoneum and muscular coat, that it is difficult precisely to determine their arrangement. From these fibres, the plexus proceeds across the uterus in the form of a thin web to the point where the spermatic vein is leaving the uterus. After closely uniting with the nerves accompanying the uterine vessels, this plexus proceeds outwards to the round ligament, becoming less firmly adherent to the peritoneum, where it unites with a plexus on the anterior surface of the uterus and spreads out into a great web under the peritoneum. This plexus is loosely attached through its whole course to the subjacent muscular coat, by soft cellular membrane.

From the second, third, and fourth sacral nerves, but chiefly from the third, branches pass into the posterior border of the ganglion at the cervix, and are lost in its mass. From the inner surface of the ganglion, numerous small white soft nerves are given off to the neck of the uterus, some of which ramify under the peritoneum and others pass deep into the muscular coat. From the anterior and inferior borders of the ganglion, many large nerves are given off to the bladder and vagina, and from its posterior margin to the rectum.

On the left side, the spermatic nerves form a plexus around the spermatic artery from about two inches from its origin. A small branch is then sent off from the spermatic artery to the ureter accompanied with some filaments of nerves. The spermatic artery then passes down between the spermatic veins, and some of the nerves leaving the artery, get on the outside of the veins and numerous filaments are observed ramifying on the coats of the veins, and also upon the absorbents, and forming loops around them. Branches of nerves are then sent to the fallopian tube, and to the ovarium, at the base of which a great plexus is formed. The spermatic nerves then appear to enlarge as they proceed towards the uterus along with the artery and veins, and in their course filaments are sent to the peritoneum, and with the veins of the ureter. Some filaments pass down along with the spermatic artery to anastomose with the nerves accompanying the uterine arteries and veins, and other branches pass to the round ligament and to the great plexus on the body of the uterus.

On the right side of the uterus the distribution of the hypogastric, spermatic, and sacral nerves does not differ essentially from that now described as seen on the left side. The form and situation of the great plexuses on the body of the uterus are, however, more distinct, and it presents the appearance of a white pearly fasciculated membrane, about a quarter of an inch in breadth, proceeding from the mesial line at right angles to the nerves accompanying the blood-vessels, across the body of the uterus, to the round ligament where it unites with a plexus on the anterior surface of the uterus. Numerous branches are sent off from the upper and lower borders of the posterior plexus to the muscular coat of the uterus. An extensive and intimate union at various points is distinctly perceptible between the branches sent off from this plexus and the branches of the nerves accompanying the uterine arteries and veins, and those which proceed from the hypogastric plexus and cervical ganglion to spread out and form a great nervous web under the peritoneum on the posterior surface of the uterus.

On the anterior and upper part of the neck of the uterus, there is a great mass of reddish coloured fibres, firmly interlaced together, resembling a thin broad ganglion of nerves, into which numerous large branches of the hypogastric nerves on both sides enter, and to which they firmly adhere. From the upper part of this fibrous substance, there passes up under the peritoneum over the whole anterior surface of the uterus a great plexus, the branches of which pass into the muscular coat, or unite with those nerves proceeding with the blood-vessels to the upper part of the uterus. Prolongations of this plexus also extend to the round ligaments, and some of its filaments unite with those of the spermatic nerves.

From the form, colour, and general appearance of these plexuses on the body of the uterus, and the resemblance they bear to ganglionic plexuses of nerves, and from their branches actually anastomosing and coalescing with the hypogastric and spermatic nerves, I was induced to conclude, on first discovering them, that they were nervous plexuses and constituted the special nervous sytem of the uterus."

3. *Nerves of the Gravid Uterus in the Ninth Month.*

Dr. Lee describes two dissections of the Gravid Uterus at this period. We shall quote the second.

" On the 12th of September, 1840, I enjoyed another opportunity of examining the nerves of the uterus at the end of the ninth month. The fœtus and its appendages had been expelled a short time before the death of the patient from whom the uterus was obtained. The spermatic nerve on both sides passed off from the renal plexus, and after accompanying the spermatic arteries for about two inches, several considerable branches left these vessels and passed to the veins which they surrounded and followed to the uterus. The absorbents on the right side were seen covered with filaments of nerves. The aortic plexus and the hypogastric nerves and plexuses presented the same appearance as in the last dissection, and were larger than in the uterus of six months. Branches were seen proceeding from the anterior part of each hypogastric plexus to the corresponding ureter, the uterine artery and vein, and to the neck and body of the uterus, as above described. The trunk of each hypogastric nerve was prolonged through the middle of the plexus to the ganglion at the cervix, into which the second, third, and fourth sacral nerves sent branches. Between the posterior part of each hypogastric plexus and the sacral nerves, there were communicating branches which did not enter the ganglion. The ganglion on each side was situated close to the neck of the uterus, a little behind the ureter near its termination in the bladder. The ganglia at the cervix appeared to be more expanded than in the former dissections, and a great communication was formed between the numerous small soft nerves passing from their inner surface and the great plexuses on the body of the uterus. From each ganglion branches spread out under the peritoneum, both of the anterior and posterior surfaces of the uterus, and many were seen plunging into the muscular coat at the cervix, and others passing up with the blood-vessels to the fundus uteri. The ureters were also observed to be surrounded with nerves from the ganglia, and many branches passed from them to the sides of the bladder, vagina and rectum. The upper borders of the great plexuses on the body of the uterus behind, were traced to a considerable depth passing in the cellular membrane between the layers of the muscular coat of the fundus uteri, which I did not perceive in the last dissection. Several trunks of absorbents on the sides of the body of the uterus were supplied with nervous filaments. The most striking circumstance observed in this dissection, was the direct continuity at numerous points between the branches proceeding from the hypogastric plexuses and ganglia at the cervix, and the branches of the great transverse plexuses on the body of the uterus behind. In the previous examination of the uterus, I had never observed filaments of nerves ramifying upon the coats of the absorbents. I have since seen this in the gravid uterus of the cow, and in the human spermatic cord, at a short distance from the testicle. Analogy led me to suspect, that many branches of nerves would also be found on examination to accompany the veins of the spermatic cord and to ramify upon their coats. Mr. James Dunn, at St. George's Hospital, undertook, at my request, to ascertain if this were the fact, and in July last he made three preparations in which the nerves are seen covering the veins as they pass out of the testicle. It appears from these, that a much greater number of nerves accompany the veins than the artery in the spermatic cord. The nerves in these preparations form a great plexus around the veins, and are traced into the testicle."

4. *Nerves of the Gravid Uterus in the Fourth Month.*

" In October, 1840, I finished the dissection of a gravid uterus of four months, all the arteries and veins on the right side of which are completely filled with red and blue injection, and the whole nervous system of the uterus more perfectly displayed than in any of the preparations already described. The uterus was removed from the body of a woman, who died in St. George's Hospital, from an external injury, and the fœtus and its appendages were expelled a few hours before death. The nerves were traced while the uterus was covered with rectified spirit. An artery of considerable size filled with injection, is seen accompanying the right hypogastric nerve, and passing along with its branches through the hypogastric plexus to the ganglion at the cervix. In this course, the artery is seen ramifying upon the trunk of the hypogastric nerve, and the most minute branches of the hypogastric plexus. The sacral nerves passing into the ganglion, are also accompanied with an artery, which is likewise injected, and which passes through the centre of the ganglion. These nerves are a little smaller than in the uteri of nine months. The ganglion is thick, large and distinct, of an oblong form, about three quarters of an inch in diameter, and consisting of grey and white matter. From its inferior border, three large bundles or masses of nervous fibres are sent off, which present an appearance resembling the pes anserinus of the portio dura. The posterior of these subdivides into numerous small branches, accompanied with arteries, which supply the rectum and back part of the vagina. The middle of these great nerves proceeding from the ganglion, likewise accompanied with arteries, ramifies upon the side of the vagina; and the anterior upon the bladder around the entrance of the ureter.

From the hypogastric plexus, before it enters the ganglion, and from the inner surface of the ganglion, numerous large and small branches of nerves are given off to the neck of the uterus, some of which accompany the blood-vessels toward the fundus, and others spread out under the peritoneum. All these are likewise accompanied by injected arteries. From the inner border of the ganglion, a broad nervous band is sent off, which passes on the outside of the ureter, and another on the inside, which unite and completely surround the ureter. From these united nervous bands, many large branches are sent to the back part of the bladder and into the anterior part of the cervix uteri. The course of these branches can easily be traced by their injected arteries. On the lower and anterior part of the cervix uteri, over the mesial line, there is a thick membranous expansion, into which these nerves enter from both hypogastric plexuses and ganglia. From the sides and upper part of this membrane, there are given off innumerable filaments, apparently nervous, which unite on the sides of the uterus with the nerves accompanying the blood-vessels, and with the spermatic nerves, and some of which pass out with the round ligaments. These are likewise accompanied with minute arteries, as all the nerves are on the right side of the uterus, entering the ganglion, and passing out from it.

The nerves and ganglion on the left side, correspond in appearance with those of the right, and the greater number of the spermatic nerves on both sides accompany the spermatic veins.

I had never before seen the arteries of the nerves of the uterus injected, or suspected that they had concomitant arteries which enlarged along with them during pregnancy."

5. *Nerves of the Uterus ten days after delivery.*

" On the 27th of June, 1840, I examined the uterus of a woman who died suddenly on the tenth day after delivery. The hypogastric plexuses, and those both on the anterior and posterior surfaces of the body of the uterus, were very much reduced in size from what they were observed to be in the uteri of six and nine months. This observation made it certain, that the nerves of the uterus after having performed their proper function during gestation and in labour, gradually return to the condition in which they are found in the unimpregnated uterus."

Two dissections are narrated. We introduce the second.

Mr. James Dunn has nearly completed a dissection of the nerves of a uterus, which was recently obtained by me at St. George's Hospital, from the body of a young woman, who had never been pregnant. The aorta, vena cava, and all the arteries and veins, connected with the uterus, have been injected. The uterus is of the ordinary size. The spermatic nerves on both sides are given off mainly by the renal plexus. The greater number of these nerves are distributed upon the veins, and not upon the spermatic arteries. Branches are sent to the fallopian tubes and ovaria, and others pass down to the uterus, and can be distinctly traced into the great transverse plexuses, under the peritoneum behind.

The aortic plexus, hypogastric nerves and plexuses are considerably larger than in the last dissection. The arteries of these nerves and plexuses have been successfully injected, and it is evident that they are much smaller, than the arteries in the gravid uterus at the fourth month. The ganglia are large and distinct, and the branches sent off to the bladder, vagina and rectum, are all accompanied by arteries filled with injection. Numerous branches of nerves are seen accompanying the uterine blood-vessels to the fundus, and the plexuses on the body of the uterus, before and behind, are continuous with the branches proceeding from the hypogastric plexuses, and the ganglia at the cervix.

These dissections prove that the human unimpregnated uterus possesses a great system of nerves, which enlarges with the coats, blood-vessels, and absorbents during pregnancy, and which returns after parturition to its original condition before conception took place. It is chiefly by the influence of these nerves, that the uterus performs the varied functions of menstruation, conception and parturition, and it is solely by their means, that the whole fabric of the nervous system sympathises with the different morbid affections of the uterus. If these nerves of the uterus could not be demonstrated to exist, its physiology and pathology would be completely inexplicable.

6. *Nerves of the Uterus in the Mare.*

There are two dense oblong ganglia in the mare, situated on the sides of the aorta a few lines above its bifurcation. The two cords of the great sympathetic nerve terminate in the superior extremities of these aortic ganglia, which are connected together by two branches of nerves, which pass across the front of the aorta from their inner borders. Each ganglion sends off a branch from its outer border, to join the spermatic nerves. From the inferior ends of these ganglia are given off the hypogastric nerves, and the nerves which supply the cornua of the uterus.

The *left hypogastric nerve* proceeds downward from the left aortic ganglion between the folds of the broad ligament, by the side of the uterus to the cervix, where it terminates in the hypogastric plexus. This nerve is at first much larger than the nerve of the cornu, and appears to be a continuation of the ganglion, and in its course it has two distinct ganglionic enlargements. The first of these is three inches from the aortic ganglion, and the second more than two inches lower down, and from both of these several large and small branches are sent off to unite with the nerve of the left cornu, and with the plexus of nerves accompanying the trunk of the uterine artery.

The *nerve of the left cornu,* soon after being given off by the aortic ganglion, divides into two branches, which again unite at a distance of three inches from the ganglion and form one nerve. This nerve suddenly expands into a firm round elongated ganglion, which resembles the bulbous root of a plant. On leaving this ganglion the nerve is enlarged to more than double the size it is on entering the ganglion, and it proceeds without any considerable diminution for four inches, to the uterine artery, with the branches of which it ramifies upon the upper part of the body of the uterus, and the whole cornu. Numerous small filaments are given off from the nerve in its course with the artery to the peritoneum and muscular coat, and it forms a great plexus of nerves, which not only surrounds the branches of the artery, but the veins and absorbents. On the nerve of the right cornu of the uterus, a round elongated ganglion is also formed, about the same distance from its origin, and the nerve passes out of the ganglion much increased in size, and is distributed to the body of the uterus and cornu, as on the left side.

From each hypogastric plexus there are sent off to the body and neck of the uterus many large nerves, which have a very peculiar serpentine or undulated appearance. They proceed between the folds of the broad ligaments to the anterior and posterior surfaces of the uterus, and after sending numerous branches to the peritoneum, they penetrate the muscular coat, and can easily be traced ramifying upon it on to the cornua. The greater number of these nerves are found on the anterior surface of the uterus, where they form an immense plexus in the muscular coat, and where they can be seen as they cross the arteries, anastomosing with the branches of the nerves of the cornua. At the cervix, these nerves can easily be traced through the muscular coat to the lining membrane of the uterus, under which they form a beautiful nervous web. These great nerves in the uterus of the mare, resemble in their origin and distribution, the ganglionic plexuses in the human uterus above described, which I regard as the muscular nerves of the organ. The aortic ganglia in the mare correspond with the aortic plexus in the human subject, and the nerves of the cornua with the branches of the hypogastric plexuses which pass down between the uterus and ureters, to the trunks of the uterine artery and vein, with the branches of which they ramify on the body and fundus of the uterus.

Dr. Lee has examined with the microscope the plexuses on the body of the human uterus, and the spermatic and hypogastric nerves, and has been unable to discover any appreciable difference between them.

Two plates accompany and illustrate the description. The first represents the nerves of the uterus in the sixth month of pregnancy—the second, those nerves in the ninth month. Both are views of the posterior surface of the uterus.

We have witnessed the preparations, which are faithfully delineated. Without offering a confident opinion on the nature of what Dr. Lee considers nerves, we can, at all events, do justice, and we ought to do it, to the patient manner in which the investigation has been conducted, and the fidelity with which what has been found has been described. Dr. Lee is still pursuing the inquiry, and we may look forward to its ultimate complete solution at his hands. It redounds infinitely to his honour.

